# Progress in the development of Reversed Chloroquine molecules as antimalarial therapy

**DOI:** 10.1186/1475-2875-9-S2-O6

**Published:** 2010-10-20

**Authors:** Steven J Burgess, Jane Xu-Kelly, Katherine Liebmann, Bornface Gunsaru, Westin Morrill, David H Peyton

**Affiliations:** 1 Department of Chemistry, Portland State University, Portland, Oregon 97207-0751, USA; 2 DesignMedix, Inc., 2828 SW Corbett Avenue, Portland, Oregon 97201, USA

## Background

Chloroquine (CQ) resistance in *P. falciparum* is strongly linked to mutations in the *gene pfcrt* that gives rise to the protein, PfCRT (*P. falciparum* chloroquine resistance transporter), located in the parasite's digestive vacuole (DV) membrane [1]. In CQ resistant (CQR) strains, accumulation of CQ is reduced in the DV due to increased efflux [2], relative to CQ sensitive (CQS) malaria.

## Method

We have developed a class of molecules, termed Reversed Chloroquine compounds (RCQs), that are hybrids made up of a chloroquine (CQ) like moiety, and a chemosensitizer (Reversal Agent, RA) against CQR in malaria [3]. Subsequent structure-activity relationship (SAR) work showed the RCQ molecular design to be very flexible. Thus, a large variety of RCQ molecules has been constructed that retain the *in vitro* efficacy against *P. falciparum* malaria [4].

## Results

The RCQ molecules have been shown to have low- to even sub-nanomolar *in vitro* IC_50_ values against both CQR and CQS malaria strains. Here we report on an expanded set of RCQ entities. A subset of these drug candidates has been tested in mouse models of malaria, and found to be capable of reducing the parasite burden to below detectable limits; being able to cure by the oral route. Furthermore, RCQ molecules are shown to have enhanced uptake into CQR parasites. Both cytotoxicity and acute toxicity in mice are favorable, as is Ames evaluation of mutagenicity. hERG binding SAR shows that variation in the RCQ structure can be used to minimize potential cardiotoxicity issues.

## Conclusion

The RCQ approach remains a strong contender to exceed the advantages that CQ brought to malaria chemotherapy for nearly half a century, before being thwarted by resistance.
